# Comparative mechanics of diverse mammalian carotid arteries

**DOI:** 10.1371/journal.pone.0202123

**Published:** 2018-08-10

**Authors:** David A. Prim, Mohamed A. Mohamed, Brooks A. Lane, Kelley Poblete, Mark A. Wierzbicki, Susan M. Lessner, Tarek Shazly, John F. Eberth

**Affiliations:** 1 College of Engineering and Computing, Biomedical Engineering Program, University of South Carolina, Columbia, SC, United States of America; 2 Cullen College of Engineering, Biomedical Engineering Department, University of Houston, Houston, TX, United States of America; 3 College of Health Sciences, Physical Therapy Program, Texas Women’s University, Houston, TX, United States of America; 4 Dwight Look College of Engineering, Biomedical Engineering Department, Texas A&M University, College Station, TX, United States of America; 5 School of Medicine, Department of Cell Biology and Anatomy, University of South Carolina, Columbia, SC, United States of America; 6 College of Engineering and Computing, Mechanical Engineering Department, University of South Carolina, Columbia, SC, United States of America; University of Michigan, UNITED STATES

## Abstract

The prevalence of diverse animal models as surrogates for human vascular pathologies necessitate a comprehensive understanding of the differences that exist between species. Comparative passive mechanics are presented here for the common carotid arteries taken from bovine, porcine, ovine, leporine, murine-rat, and murine-mouse specimens. Data is generated using a scalable biaxial mechanical testing device following consistent circumferential (pressure-diameter) and axial (force-length) testing protocols. The structural mechanical response of carotids under equivalent loading, quantified by the deformed inner radius, deformed wall thickness, lumen area compliance and axial force, varies significantly among species but generally follows allometric scaling. Conversely, descriptors of the local mechanical response within the deformed arterial wall, including mean circumferential stress, mid-wall circumferential stretch, and mean axial stress, are relatively consistent across species. Unlike the larger animals studied, the diameter distensibility curves of murine specimens are non-monotonic and have a significantly higher value at 100 mmHg. Taken together, our results provide baseline structural and mechanical information for carotid arteries across a broad range of common animal models.

## Introduction

In humans and all other mammals, common carotid arteries (CCAs) supply oxygenated blood to critical cognitive and masticatory structures. Atherosclerosis of the CCAs, otherwise known as carotid artery disease, begins as an asymptomatic pathology and can lead to stenosis, plaque embolism, and ischemic stroke [1]. Of approximately 800,000 strokes in the U.S. each year as many as 15% can be attributed to carotid artery disease [2,3]. According to a recent clinical investigation, over 30% of asymptomatic patients aged 40–54 exhibit subclinical CCA atherosclerosis [4], suggesting a very large aging population at future risk of stroke and motivating further study into disease progression, diagnosis, and treatment.

Mechanical changes in carotid arteries can occur as a result of surgical intervention, device implantation, local and systemic disease progression, and tissue remodeling in response to altered hemodynamic stimuli. For example, carotid arteries from patients with dyslipidemia and type II diabetes were shown to be stiffer than paired noninjured arteries [5], which is commonly acknowledged as a predictor of increased risk of mortality [6]. Carotid artery stenting and carotid endarterectomy on the other hand, are the two most common interventions for severe carotid artery disease [7]. In either case understanding the baseline CCA mechanics can be fundamental to characterizing both healthy remodeling and undesirable pathologies [8,9].

Animal models are commonly used as human surrogates to test interventional devices and novel therapies. For studies involving CCA mechanics, it may be prudent to select a scalable model with specific baseline values of structural or mechanical properties under conditions of interest, and a comprehensive understanding of how animal-specific tissues behave under diverse biaxial loading scenarios are paramount to the translatability of such a study [9–16]. Despite this, it is not clear which CCA properties follow allometric scaling laws thereby hindering the initial screening of the diversity of potential candidates [17–20].

In this study, we investigate and compare the passive mechanical properties of CCAs freshly harvested from bovine, porcine, ovine, leporine, and two murine animals, the laboratory rat and the laboratory mouse. A unique yet modular biaxial testing device is used to measure the passive mechanical properties through inflation-extension testing using similar protocols and procedures for each vessel. Obtained results therefor provide a catalog of information that is used to determine scalability in order to guide the selection of animal models for various CCA studies.

## Materials and methods

### Vessel isolation and preparation

Male bovine (Angus cow; 500 kg), porcine (American Yorkshire pig; 108 kg), ovine (Suffolk-Rambouillet sheep; 35.1 kg), and leporine (New Zealand White rabbit; 4.03 kg) carotid specimens (n = 6; each) were obtained from abattoirs at roughly 18, 6, 10, and 3 months old respectively. Specimens from bovine, porcine, ovine, and leporine animals were designated for consumption and considered to be mature at the time of slaughter. These specimens were obtained from three local abattoirs with the following approximate geographic coordinates: (30.028, -95.361), (29.786, -95.975), and (29.675, -95.618). The ages of the bovine, porcine, ovine, and leporine specimens were selected based on their highest availability and recommended age for slaughter. Murine animals on the other hand, were selected at an age of maturity that roughly compares to the abattoir counterparts so that all animals experience a similar number of heartbeats in their lifetime, which we consider to represent a similar level of cardiovascular maturity. Laboratory murine-rat (Sprague-Dawley rat; 0.49 kg) and murine-mouse (C57BL/6J mouse; 0.028 kg) animals were sacrificed using C0_2_ inhalation at 8 and 3 months of age respectively. All animal procedures were approved by the Animal Care and Use Committees (IACUC) at the University of Houston and the University of South Carolina.

Vessels were harvested immediately after slaughter/sacrifice and transported in sterile Moscona’s saline solution at 4° C to the laboratory where perivascular and loose adventitial tissue was removed from each sample. The perivascular tissue was notably more difficult to remove from the larger vessels. To minimize tissue mounting edge effects, vessels were trimmed so that the length was at least 10-times greater than the diameter but fit within the 25 cm long tissue bath of the biaxial testing device once loaded. Vessels were then secured to appropriately-sized barbed fittings, syringe needles, or glass cannulas (mouse only) with braided silk suture. Prior to and throughout testing, Krebs-Henseleit solution at pH 7.4 and containing 10^−5^ M sodium nitroprusside was perfused through, and around, the sample and then continuously circulated in the adventitial bath in order to guarantee a fully relaxed smooth muscle state. Once mounted within the test chamber samples were maintained at 37.2 ^o^C using a 15W submersion heater within the adventitial bath.

A set of specimens designated only for histological staining were perfused with 4% paraformaldehyde, pressurized to 100 mmHg, and submerged in an adventitial bath also containing 4% paraformaldehyde. These vessels were then embedded in paraffin and prepared for sectioning. Sections 5 μm thick were cut using a microtome and stained with Hematoxylin & Eosin. Stained sections were digitally imaged on a Nikon E600 microscope (Nikon Instruments, Melville, NY) using 10x, 20x, 40x, or 100x objectives and QCapture imaging software (QImaging, Surrey, BC). Oil immersion lenses were used for the murine-rat and murine-mouse specimens (100x objective).

### Mechanical testing

All mechanical testing was performed on a modular, custom-designed, biaxial mechanical testing device with LabView-based data acquisition (National Instruments; Austin TX). Reference [21] provides much of the general layout of a similar testing apparatus with hardware in that publication specific to the mouse carotid arteries and ours designed to accommodate larger specimens. Other differences in hardware or configuration are described here. Luminal inflation was initiated with a feedback controlled syringe pump (World Precision Instruments; Sarasota, FL) and pressure measured using an amplified 0–300 mmHg USB pressure transducer (Omega Engineering, Norwalk, CT). To accommodate large variations in vessel size, the objective lenses for a CMOS Monochrome 1280x1024 USB camera (Edmund Optics; Barrington, NJ) were selected to yield 580 pixels/mm, 287 pixels/mm, 144 pixels/mm, or 52.0 pixels/mm. Likewise force transducers (Omega Engineering) capable of measuring up to 0.1 N, 1.1 N, or 44 N were interchanged once a preliminary force range was determined for each species. The force transducer was connected to the blood vessel using a pivoting L-shaped arm allowing the transducer to be isolated from the liquid media. Stepper motor micrometer-driven translation stages (Thorlabs; Newton, NJ) were also chosen with 10 mm, 25 mm, or 100 mm of travel to provide adequate axial extensions. Each piece of hardware was extensively calibrated prior to data collection.

**Fig 1:**
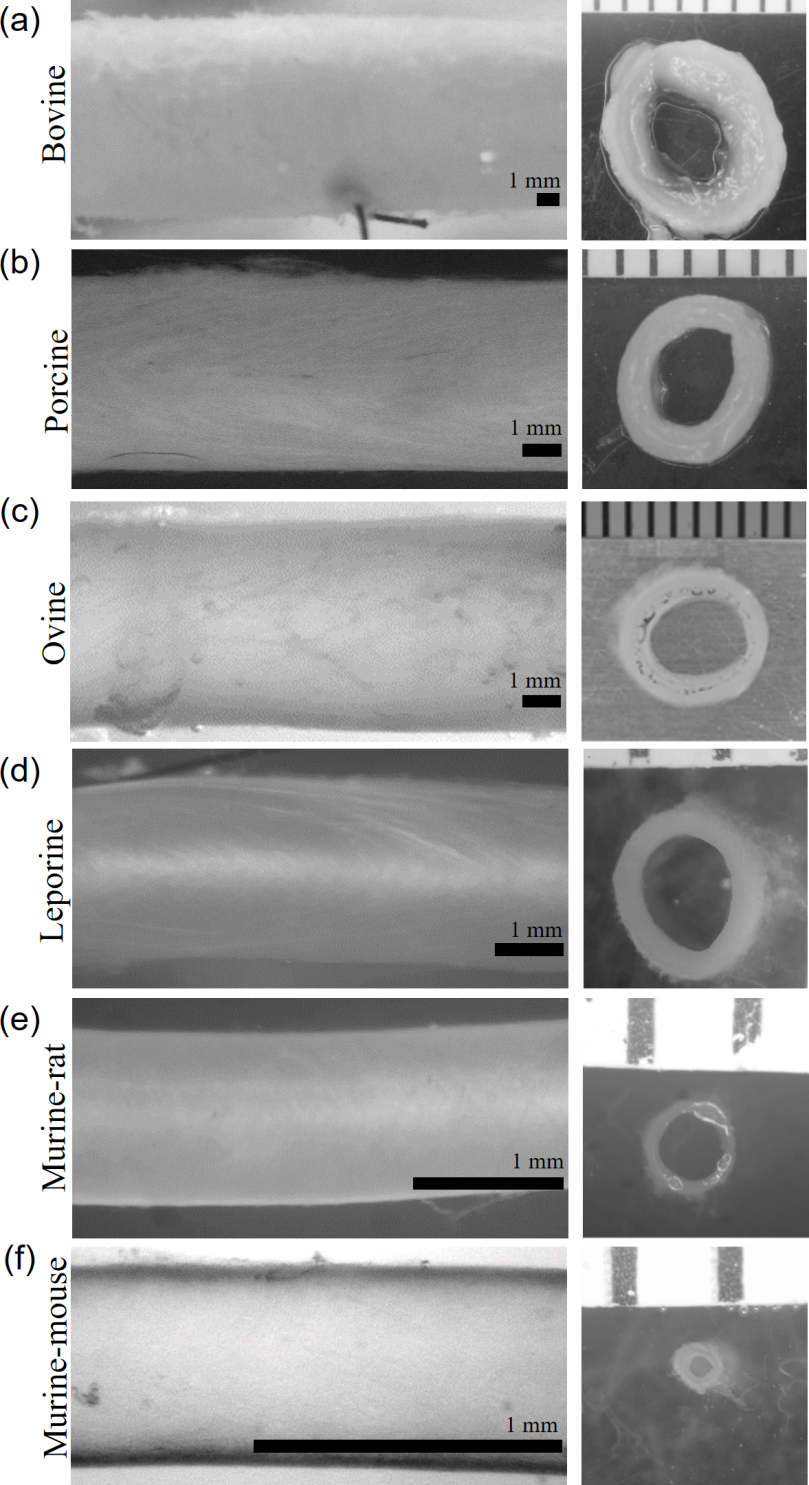
Images of common carotid arteries. (left) Vessels mounted within the biaxial testing device at λ_z_ = 1.5 and P = 100 mmHg. (a) Bovine: OD = 8.82 mm, (b) porcine: OD = 4.96 mm, (c) ovine: OD = 5.49 mm, (d) leporine: OD = 2.60 mm, (e) murine-rat: OD = 1.14, and (f) murine-mouse: OD = 0.65 mm vessels shown. Scale bars are 1 mm. (right) Unloaded ring sectors of each vessel with 1 mm ruler.

Initial measurements were made of the unloaded length and diameter with the specimen mounted in the biaxial testing device, but prior to pressurization or axial extension. To ensure repeatable loading curves, all vessels underwent three cycles of preconditioning consisting of inflation/deflation pressurization from 0–160 mmHg (1 mmHg/s) at 1.50±10% axial extension ratio. Following preconditioning, vessels were axially extended to 1.5 and pressurized step-wise from 0–160 mmHg in 10 mmHg increments. For circumferential testing (pressure-diameter) a 10 second equilibrium period was allowed at each step then diameter measurements were collected via the calibrated CMOS camera (Fig 1, left). Next, luminal pressure was adjusted to 100 mmHg, and the axial stretch *λ*_*z*_ set so that vessels experienced a very small tensile force. This stretch was recorded as $\lambda_{z}^{\min}$. For axial testing (force-length) vessels were extended axially in stretch increments corresponding to a 0.05 increase in extension ratio while recording axial force at each step. These two mechanical testing regimes were selected based on a known configuration that prevents buckling (roughly circumferential testing at *λ*_*z*_ = 1.5) and similar in vivo arterial pressures amongst mammals (axial testing at P = 100 mmHg).

### Data analysis

Vessels were removed from the device and centrally located ring sections cut from the samples. Images of rings were collected using a microscope mounted DSLR camera (Canon USA, Long Island NY) and the magnification set to maximize the field of view using the millimeter scale of a ruler (Fig 1, right). The unloaded inner and outer circumference of each section were quantified using ImageJ software (NIH), and the inner *R*_*i*_ and outer *R*_*o*_ radii calculated. From this, the cross-sectional tissue area was calculated via

$$A=\pi (R_{o}^{2}-R_{i}^{2}).$$ (1)

Unlike prior work (see [12,22]), we chose not to induce a stress relieving cut. Although straightforward with large vessels, prior experience with mouse and rat carotid arteries suggested that the stress relieving cut would introduce considerable error (unpublished laboratory observations).

Through real time measurements of outer diameter and assuming vessel incompressibility, the deformed inner radius *r*_*i*_ is calculated from

$$r_{i}=\sqrt{r_{o}^{2}-\frac{A}{\pi \lambda_{z}}},$$ (2)

where *r*_*o*_ = *OD*/2 is the deformed outer radius and *λ*_*z*_ the axial stretch ratio. The lumen area compliance and diameter distensibility are

$$C_{A}=\pi \frac{\Delta r_{i}^{2}}{\Delta P}\;\mathrm{and}\;D_{d}=2\frac{\Delta d_{i}/d_{i}}{\Delta P},$$ (3)

where Δ*P* is the change in transmural pressure. For compliance (mm^2^/mmHg), Δ*P* is measured around 100 mmHg and Δ*r*_*i*_ the change in radius about this point. Distensibility (mm^2^/mmHg), on the other hand, is a normalized metric useful for comparing mechanical properties of vessels from different species [23]. Mean circumferential and axial stresses are calculated as

$$\sigma_{\theta }=\frac{{Pr}_{i}}{h},\;\sigma_{z}=\frac{F+P\pi r_{i}^{2}}{\pi h(2r_{i}+h)}$$ (4)

with *F*, the axial force, and *h* = *r*_*o*_−*r*_*i*_ the wall thickness. The pressure contribution on the right-hand-side of Eq (4) is consistent with a force balance of a pressurized, closed tube [24].

The circumferential and axial stretches are calculated by

$$\lambda_{\theta }=\frac{r_{i}+r_{o}}{R_{i}+R_{o}},\;\lambda_{z}=\frac{l}{L},$$ (5)

where we note the radial stretch is found via incompressibility and circumferential stretch represents the mid-wall value.

### Statistical analysis

**Fig 2:**
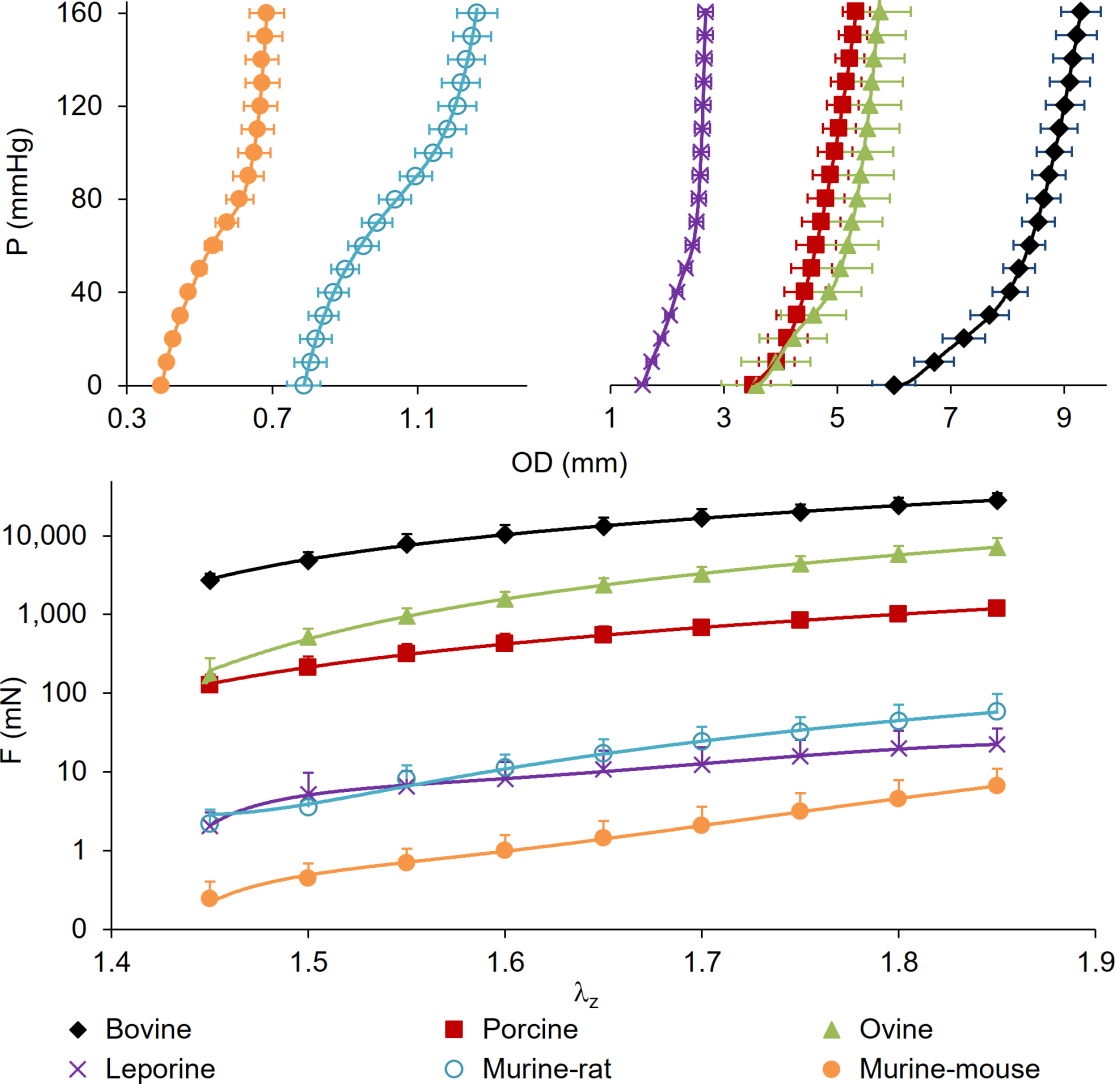
Full range of common carotid arteries subjected to passive mechanical testing. (top) Pressure-diameter at λ_z_ = 1.5, (bottom) axial force-stretch on a logarithmic scale at P = 100 mmHg for Bovine, porcine, ovine, leporine, murine-rat, and murine-mouse. All values are mean (n = 6) ± SEM.

**Fig 3:**
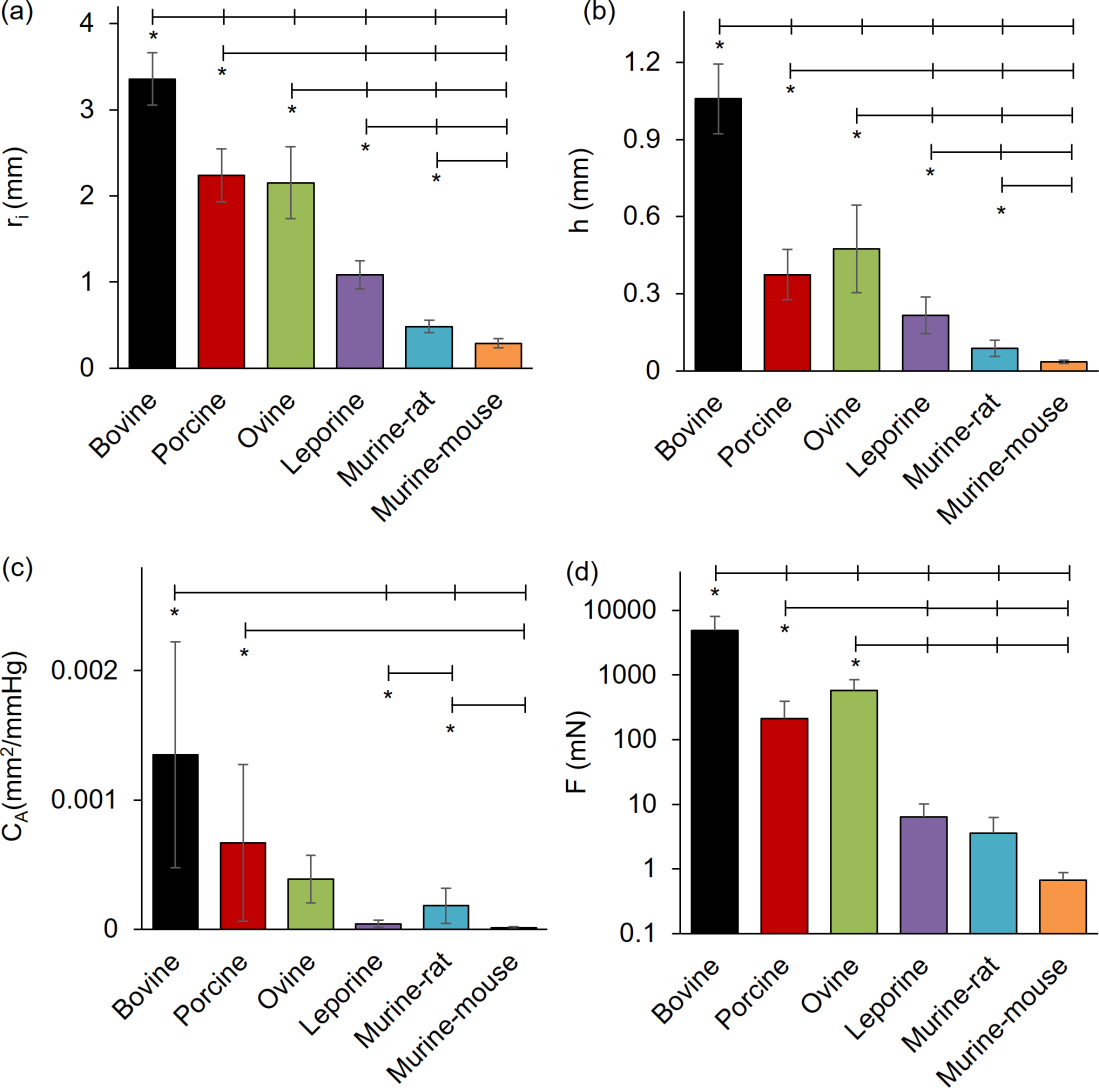
Comparative structural and force values for common carotid arteries subjected to passive mechanical testing at 100 mmHg and 1.5 axial stretch ratio. (a) Inner radius, (b) wall thickness, (c) area compliance, and (d) axial force from bovine, porcine, ovine, leporine, murine-rat, and murine-mouse carotid arteries. All values are mean ± STD. (*) denotes statistical significance at p ≤ 0.05 between the leftmost group and the corresponding hash-mark.

Figures demonstrating continuous data (e.g., Fig 2) are expressed as the mean ± the standard error of the mean (SEM) while discrete data (e.g., Fig 3) are presented as the mean ± the standard deviation (STD). Paired comparisons were performed using two-tailed student t-tests with a value of p ≤ 0.05 considered to be significant.

## Results

Data is displayed for bovine, porcine, ovine, leporine, murine-rat, and murine-mouse groups in the order of decreasing body weight with values representing the mean of n = 6 samples. Most measured structural quantities are shown to increase with animal weight. The pressure-outer diameter graph shows similar patterns within physiological ranges across the six species tested (Fig 2, top). Notable differences include the murine carotid samples that have a concave pattern at pressures below 80 mmHg but transitions to a convex relationship at higher pressures, while porcine, ovine, leporine, and bovine samples display a convex relationship at all tested pressures.

When compared at 100 mmHg and 1.5 axial stretch ratios (Fig 3) the inner radius and wall thickness, of bovine (*r*_*i*_ = 3.35 ± 0.30 mm, p<0.001; *h* = 1.06 ± 0.14 mm, p<0.001), porcine (*r*_*i*_ = 2.24 ± 0.31 mm, p<0.001; *h* = 0.37 ± 0.10 mm, p<0.001), and ovine (*r*_*i*_ = 2.15 ± 0.42 mm, p<0.004; *h* = 0.48 ± 0.17 mm, p<0.016) arteries were significantly different than those of leporine (*r*_*i*_ = 1.08 ± 0.16 mm; *h* = 0.22 ± 0.07 mm), murine-rat (*r*_*i*_ = 0.484 ± 0.070 mm; *h* = 0.087 ± 0.031 mm), or murine-mouse (*r*_*i*_ = 0.290 ± 0.070 mm; *h* = 0.035 ± 0.001 mm). Despite a high standard deviation in lumen area compliance, differences were still significant for bovine (*C*_*A*_ = 1.5x10^-3^ ± 8.1x10^-4^ mm^2^/mmHg, p<0.004) compared to leporine (*C*_*A*_ = 6.1x10^-5^ ± 3.9x10^-5^ mm^2^/mmHg; p = 0.022), murine-rat (*C*_*A*_ = 3.2x10^-4^ ± 1.3x10^-4^ mm^2^/mmHg, p = 0.040), or murine-mouse (*C*_*A*_ = 2.6x10^-5^ ± 1.9x10^-5^ mm^2^/mmHg, p = 0.021). Significant area compliance differences were also found between porcine (*C*_*A*_ = 6.8x10^-4^ ± 5.6x10^-4^ mm^2^/mmHg) and murine-mouse (p = 0.047), and between the leporine and murine-rat (*C*_*A*_ = 3.2x10^-4^ ± 1.3x10^-4^ mm^2^/mmHg, p = 0.004). Average values of axial force and axial stretch also display similar, but highly nonlinear relationships across species that are more evident on a non-logarithmic scale. The axial force for the bovine specimens (*F* = 4,890 ± 3,132 mN, p<0.019) was different than porcine (*F* = 213.1 ± 179.8 mN), ovine (*F* = 576.3 ± 276.7 mN), leporine (*F* = 6.430 ± 3.73 mN), murine-rat (*F* = 3.610 ± 2.62 mN) or murine-mouse (*F* = 0.693 ± 0.238 mN). The porcine and ovine specimens were also different from the leporine and murine specimens (p<0.049).

**Fig 4:**
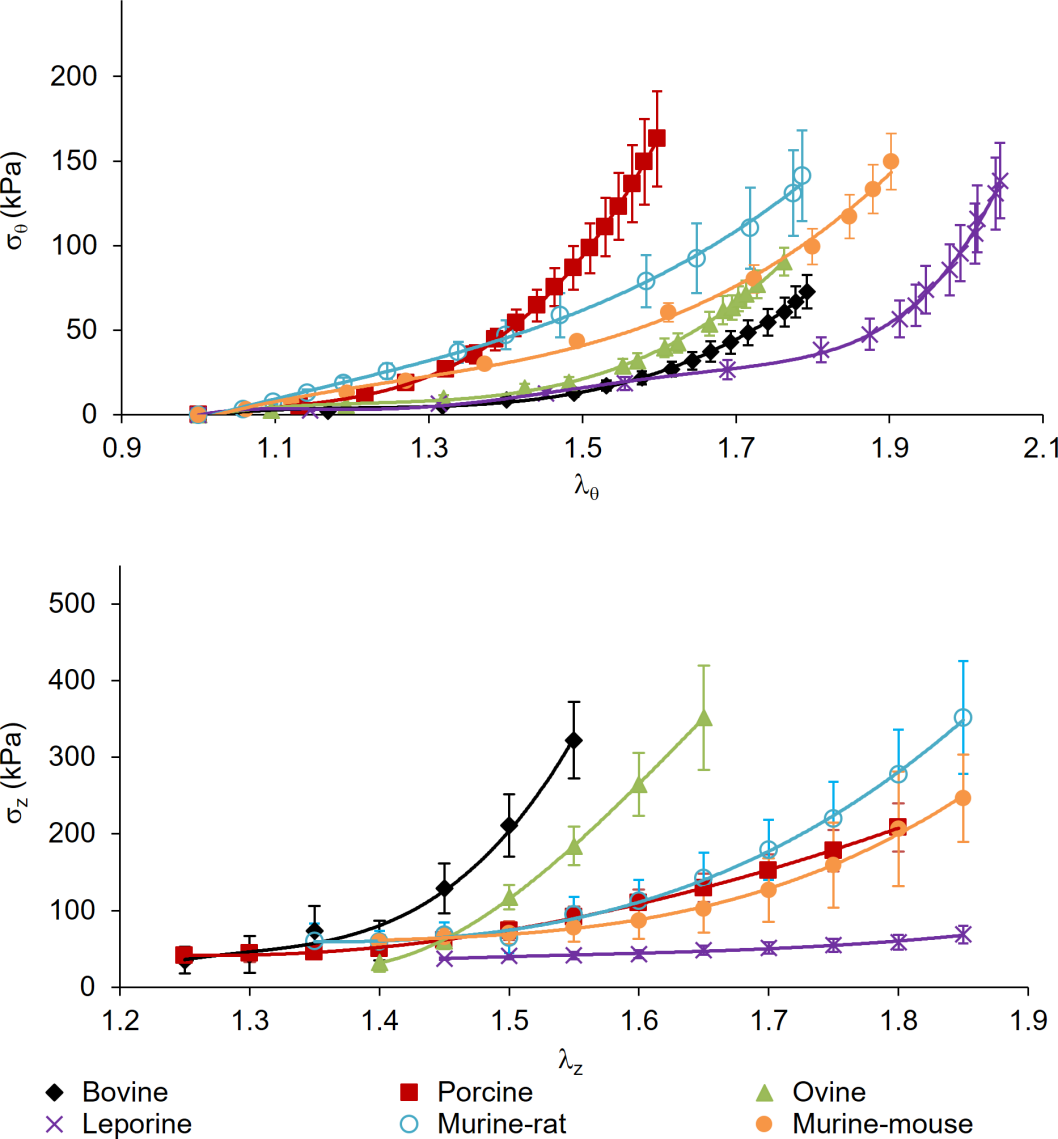
Full range of stress and stretch for common carotid arteries subjected to passive mechanical testing. (top) Circumferential stress-stretch at λ_z_ = 1.5, and (bottom) axial stress-stretch at 100 mmHg for bovine, porcine, ovine, leporine, murine-rat, and murine-mouse common carotid arteries. All values are mean (n = 6) ± SEM.

**Fig 5:**
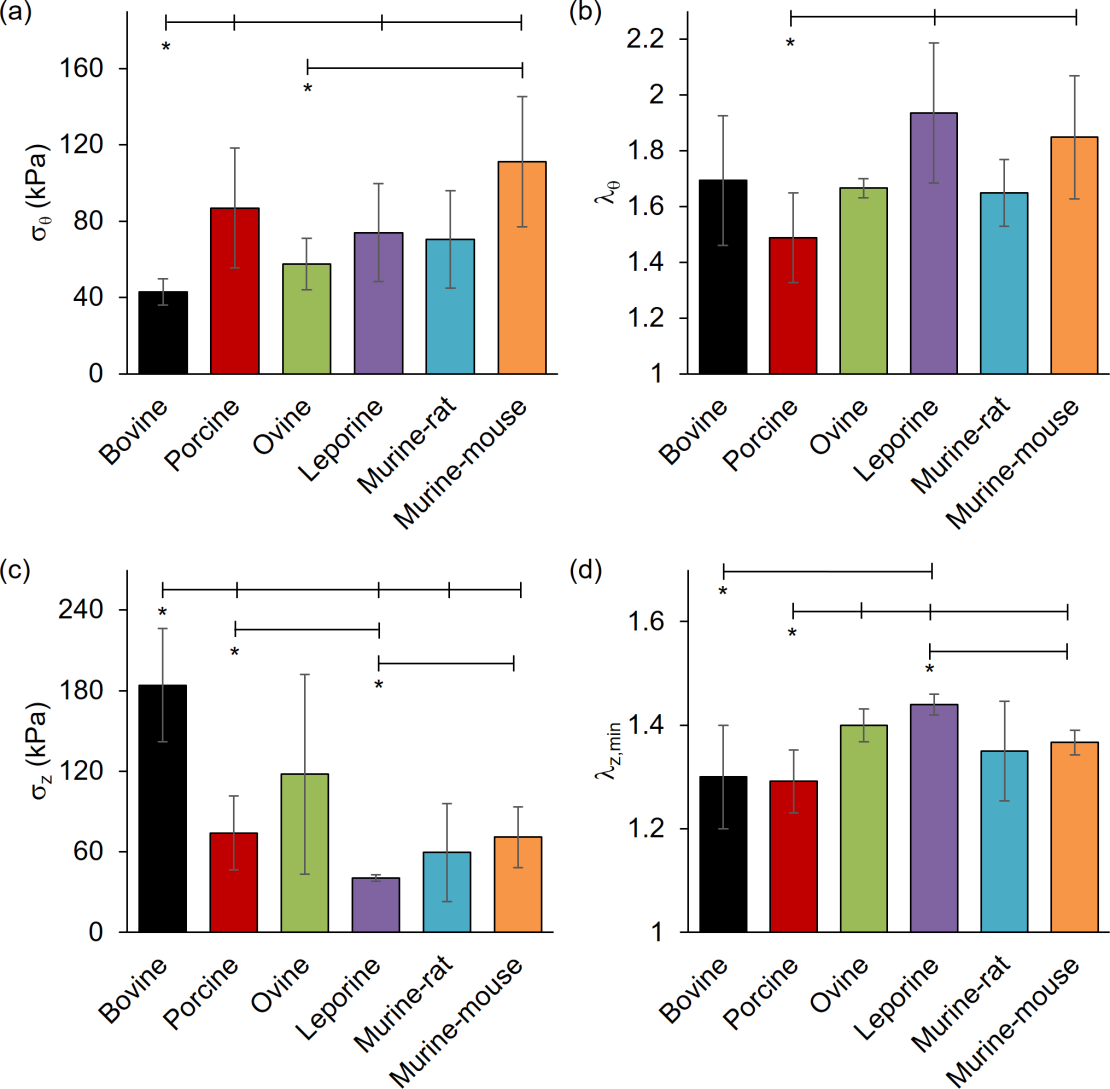
Comparative stress and strain values for common carotid arteries subjected to passive mechanical testing at 100 mmHg. (a) Circumferential stress, (b) circumferential stretch, and (c) axial stress for bovine, porcine, ovine, leporine, murine-rat, and murine-mouse carotid arteries at 1.5 axial stretch ratio. Figure (d) illustrates the minimal axial stretch ratio to maintain vessels in tension at 100 mmHg. All values are mean ± STD. (*) denotes statistical significance at p ≤ 0.05 between the leftmost group and the corresponding hash-mark.

While vessel geometry varies widely between species, the stress and stretch metrics facilitate comparisons that are independent of length scale (Fig 4 and Fig 5). Circumferential stress was found to be lower in the bovine (*σ*_*θ*_ = 42.9 ± 6.88 kPa) than porcine (*σ*_*θ*_ = 86.8 ± 31.4 kPa, p = 0.025), leporine (*σ*_*θ*_ = 74.0 ± 25.7, p = 0.041), and murine-mouse (*σ*_*θ*_ = 111 ± 34.1 kPa, p = 0.015) counterparts while ovine specimens *σ*_*θ*_ = 53.9 ± 14.2 kPa, p = 0.038) had significantly lower circumferential stress only in comparison to murine-mouse (p = 0.032). Likewise, bovine axial stress (*σ*_*z*_ = 184 ± 42.2 kPa) was higher than in porcine (*σ*_*z*_ = 74.1 ± 27.4 kPa, p = 0.049), leporine (*σ*_*z*_ = 40.6 ± 2.46 kPa, p = 0.042), murine-rat (*σ*_*z*_ = 54.3 ± 34.3 kPa, p = 0.029) and murine-mouse (*σ*_*z*_ = 70.87 ± 22.5 kPa, p = 0.050) arteries, while the porcine (p = 0.041) and murine-mouse (p = 0.029) groups were higher than the leporine group. The porcine circumferential and minimum axial stretches (*λ*_*θ*_ = 1.49 ± 0.16; *λ*_*z*,min_ = 1.29 ± 0.06) were found to be lower than leporine (*λ*_*θ*_ = 1.93 ± 0.25, p = 0.044; *λ*_*z*,min_ = 1.43 ± 0.02, p = 0.039) and murine-mouse (*λ*_*θ*_ = 1.85 ± 0.22, p = 0.016; *λ*_*z*,min_ = 1.37 ± 0.02, p = 0.039) while the minimum ovine axial stretch ratios (*λ*_*z*,min_ = 1.40 ± 0.03, p = 0.009) were higher than in the porcine specimens. The minimum axial stretch ratio was also found to be lower in the bovine (*λ*_*z*,min_ = 1.30 ± 0.10, p = 0.030) and the murine-mouse (*λ*_*z*,min_ = 1.37 ± 0.02, p = 0.032) compared to the leporine.

**Fig 6:**
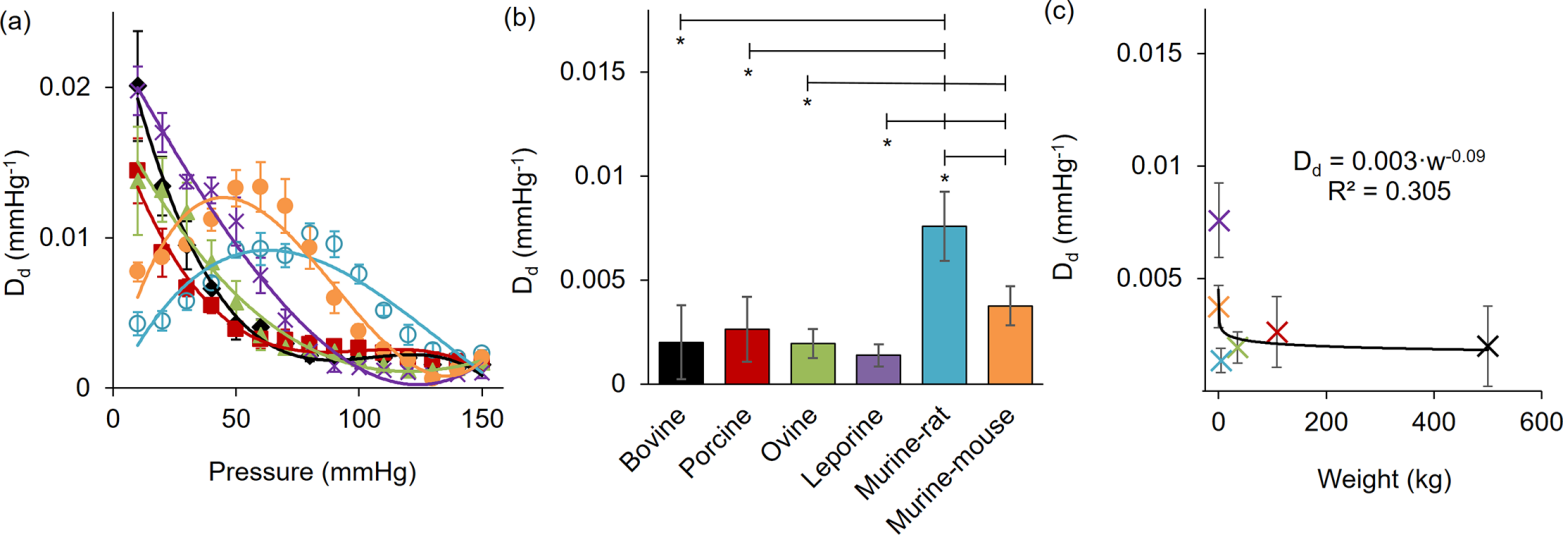
Distensibility of bovine, porcine, ovine, leporine, murine-rat, and murine-mouse carotid arteries. Diameter distensibility at (a) full pressure range mean ± SEM (b) 100 mmHg mean ± STD, and (c) 100 mmHg mean ± STD vs. animal weight and fit to a power law allometric scaling relationship with the coefficient of determination shown. (*) denotes statistical significance at p ≤ 0.05 between the leftmost group and the corresponding hash-mark.

The diameter distensibility for all carotid arteries are reported in Fig 6. Plotting these values for the full range of pressures illustrates two distinct behaviors. Namely larger animals have a monotonically decreasing distensibility with pressure while both murine animals exhibit non-monotonic behavior reaching a peak in sub physiologic pressures. Comparing distensibility around a common pressure of 100 mmHg shows a significantly higher diameter distensibility in the murine-rat (*D*_*d*_ = 7.66 x10^-3^ ± 1.65 x10^-3^ mmHg^-1^; p<0.001) than all other animals. Likewise, the murine-mouse distensibility (*D*_*d*_ = 3.76 x10^-3^ ± 9.37 x10^-4^ mmHg^-1^) was higher than the ovine (*D*_*d*_ = 1.94 x10^-3^ ± 6.99 x10^-4^ mmHg^-1^; p = 0.015) and leporine (*D*_*d*_ = 1.38 x10^-3^ ± 5.41 x10^-4^ mmHg^-1^; p = 0.001) specimens. Despite these observations, a predictable pattern could not be discerned when distensibility was plotted versus the animal weights (Fig 6C).

## Discussion

The current work reports on the mechanical properties of common carotid arteries from 6 distinct but common mammalian species. Due to the long, unbranched, and paired anatomy, CCAs, especially canine models, are well studied in the literature. Although collectively many datasets are available from a wide-variety of sources, challenges in scalability have previously been a limitation on such a wide range of being reported in a single published work. An exception to this is the experimentation performed by RH Cox (1978) who studied the mechanics of the canine, leporine, and murine-rat carotid arteries [20]. That investigation demonstrated similar outer diameter and circumferential stress to ours but no axial forces were reported. Cox, as in our study, reported a concave pattern in the pressure-diameter curves at low levels of pressure in the smaller animals but not in the larger ones. These patterns seemingly contribute to altered distensibility behaviors for smaller vessels (Fig 6). Similar patterns and diameter ranges were also reported in the work of Weizsäcker et al. (1983) who investigated biaxial properties of murine-rat carotid arteries [25]. Chuong and Fung (1983), and later Takamizawa (2015), experimented on leporine arteries and fit their results to constitutive models [10,26]. Although deformed inner radii in that latter study is similar to our current work and that of Weizsäcker et al., our reported axial forces are lower in the leporine and murine-rat models. This could be attributed to variations in an animal’s genetic background or age [27,28].

Despite the abundance of canine data, and to a lesser extent porcine, (e.g., [20,22]), there are surprisingly few inflation-extension tests of bovine carotid mechanics. Von Maltzahn (1984) conveniently performed biaxial testing on carotids samples at the same pressures and axial extensions used in our study. Although the diameters were slightly larger than those reported here, axial forces were lower [29]. These differences may be due to the breed and/or the age of the animal, but that specific information was not reported in their work. To our knowledge, Blondel and colleagues (2001) present the only published biaxial inflation-extension mechanics data to date on sheep (ovine) carotid arteries [30]. Their animals and corresponding structural parameters appear to be slightly larger than those presented here, and again, the axial force and stress data were not reported. Nevertheless, other comparable mechanical parameters appear to be consistent with the ovine results reported herein. The lack of information for bovine and ovine models is surprising considering the growing importance of these animals to the field of decellularized vascular grafts and implant models [31,32]. Of further note, all vessels appear to be less stiff than the limited existing data on human CCA mechanics [33,34].

An abundance of biaxial data from control, surgically altered, and genetically modified murine-mouse models have been reported in our prior work [16,24,28,35,36]. The carotid arteries studied in the current investigation are comparable in mechanical properties to many of the controls of those prior studies. One notable exception is that the axial stretch ratio is lower here and actually represents a sub-physiological level for the murine-mouse. In fact, it is likely that the leporine and both murine models are compared at sub-physiological levels of axial extension while the bovine models are at supra-physiological levels. Since axial extension affects virtually all our quantified metrics (see for example, eqs (2) and (5)), this condition would help to explain the high measured force (and axial stress) in bovine specimens. To eliminate additional confounding variables however, we chose to compare these species at a common axial stretch ratio while recording the minimum axial stretch required to achieve tension while pressurized. An axial stretch of 1.5 appeared to be the most common reference point from the literature for diverse mammalian species [10,22,25,37].

**Fig 7:**
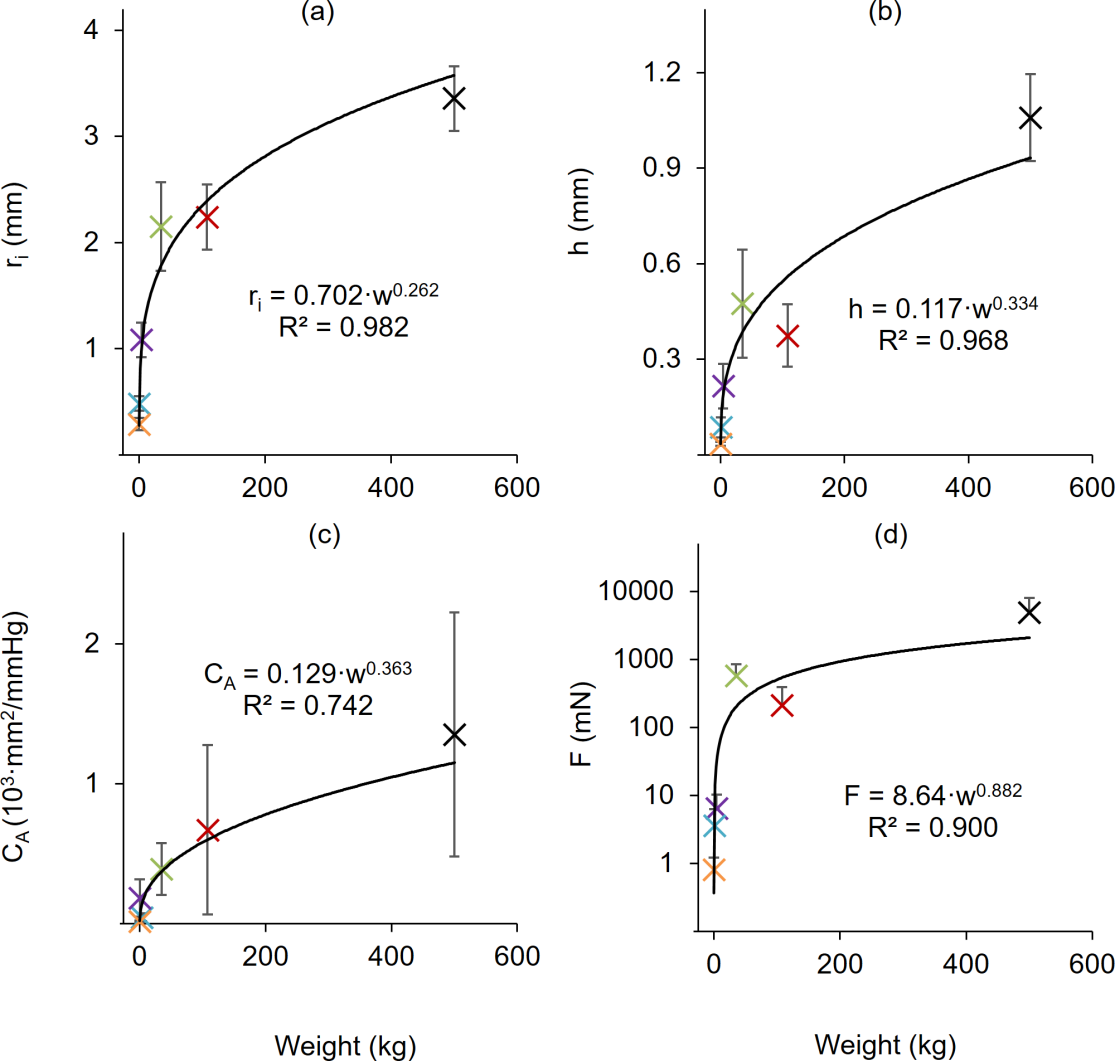
Allometric scaling of structural quantities with weight. (a) Inner radius, (b) wall thickness, (c) area compliance, (d) axial force, at 100 mmHg and λ_z_ = 1.5. All data fit to a power law allometric scaling relationship with the coefficient of determination shown. All values are mean ± STD.

**Fig 8:**
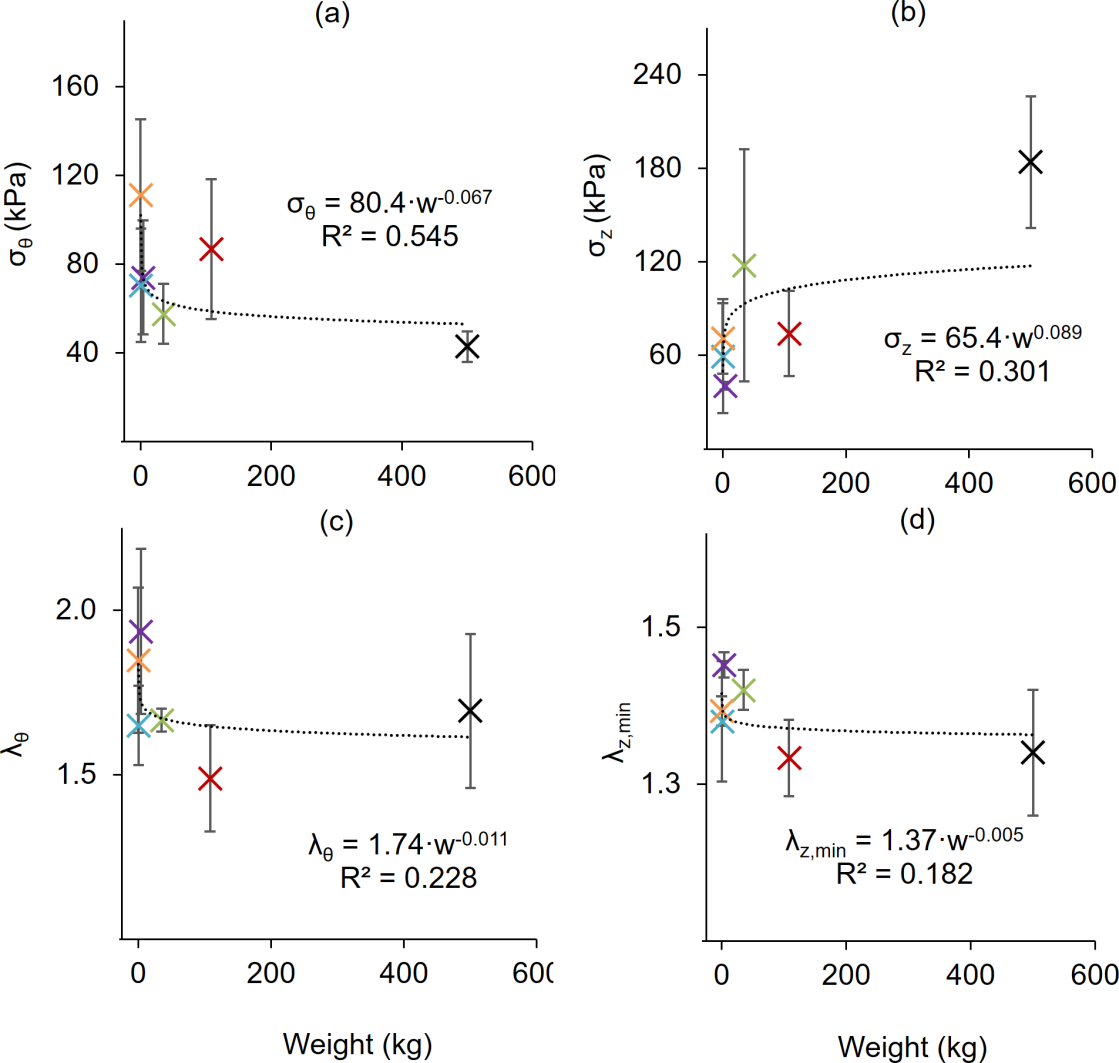
Allometric scaling of mechanical quantities with weight. (a) Circumferential stress, (b) axial stress, and (c) circumferential stretch measured at λ_z_ = 1.5. (d) Minimum axial stretch at 100 mmHg. All data fit to a power law allometric scaling relationship with the coefficient of determination shown. All values are mean ± STD.

Allometric scaling is applied to the results (Fig 7 and Fig 8, also see [38]) using a power law equation [39] of the form

$$y=k\cdot w^{a}$$ (6)

that relates the dependent variable *y* to the independent variable *w* (in this case animal weight) using a coefficient *k* and scaling exponent *a*. When plotted against body weight, the inner radius (R^2^ = 0.982), wall thickness (R^2^ = 0968), area compliance (R^2^ = 0.742), and axial force (R^2^ = 0.900) were all well represented by (6). In contrast, no obvious scaling existed for the circumferential and axial stresses and stretches or distensibility. With the exception of distensibility in murine specimens, these values, however, were closely grouped amongst many of the animals tested, but still isolated differences exist (e.g., Bovine stresses Fig 5A and 5C). Many of these differences would likely be explained by a more detailed histological study.

**Fig 9:**
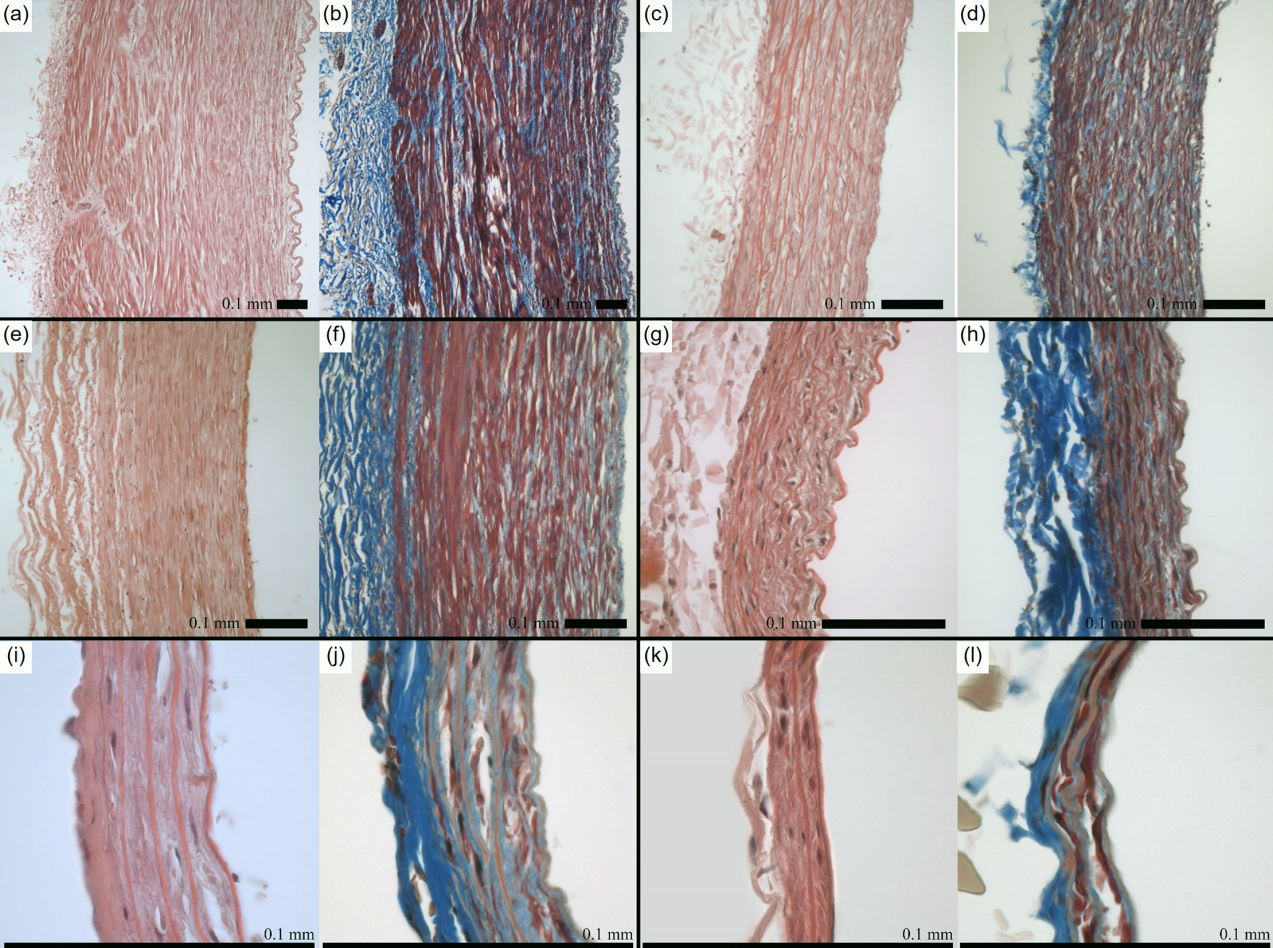
Basic histology of common carotid arteries. Hematoxylin and Eosin (left) and Masson’s Trichrome (right) for (a-b) Bovine, (c-d) porcine, (e-f) ovine, (g-h) leporine, (i-j) murine-rat, and (j-k) murine-mouse. 0.1 mm scale bar.

Wolinsky and Glagov compared aortas from diverse mammalian species to demonstrate that lamellar units–a major contributor of mechanical properties in arteries–remain relatively consistent across species [38]. These authors observed ranges in deformed outer diameter from 1.2 to 23.0 mm and body weight from 0.028 to 200 kg; despite this, aortas across all species exhibit medial lamellar units of similar size (0.006 to 0.018 mm) that experience similar tension per lamellar unit (1,090 to 3,010 dynes/cm) [38]. Moreover, the endothelial, smooth muscle, and fibroblasts cells which serve as both sensor and effector in the assembly of wall constituents, remains the same size even in a vessel wall that can be 15-fold larger. We also consider that proteins (e.g., collagen and elastin) can vary in fractional mass but their structure is preserved across species. Our H&E and Masson’s Trichrome stained cross-sections show similarities in tissue morphology, most notably a relatively thin collagenous adventitial layer and interspersed collagen fibers within the media surrounded by smooth muscle cells (Fig 9). The relative size of the cell nuclei and varying number of lamellae are readily apparent when comparing between the species at these different magnification levels that likely contribute to the certain differential mechanical responses. In line with the prior work of Wolinsky and Glagov in the aorta, the number of lamellae varies significantly across species, with a maximum of 59.7±7.6 in the bovine carotid and a minimum of 3.3±0.6 in the murine-mouse carotid; however, the average lamellar thickness is relatively similar across species (0.011 to 0.022 mm), which is also in line with prior work [38]. Although there is an intimate relationship between histology and mechanics this investigation focuses primarily on the biomechanical analysis, while future work would benefit from more thorough comparison of CCA microstructure across species.

Comparing the mechanical properties of CCAs across mammalian species supports two discrete research directions. First, animal studies are a critical tool in elucidating disease progression, evaluating novel treatment options, and developing implantable devices for a wide range of diseases, including carotid artery disease. As discussed previously, the mechanical properties of the vasculature are subject to change as a result of both disease and treatment, and quantifying these changes requires a thorough characterization of baseline mechanical properties. Age, for example, has been shown to dramatically influence arterial mechanics and would likely have a substantial effect in our study, considering the subject matter with such varied heart rates [40]. To try and avoid confounding factors from age, we used tissues with the highest availability from the abattoir and developmentally matched our laboratory specimens, resulting in samples that had experienced approximately equal number of heartbeats in their lifetime. Based on the widely examined theory that mammals experience a similar number of heartbeats regardless of life expectancy, we consider our samples to therefore have reached a similar level of cardiovascular maturity [19]. Ultimately this investigation provides preliminary mechanical data that can be used to guide the selection of animal models for carotid artery research and to provide comparative data from healthy vessels to compare with treatments and disease states.

Next, as demand continues to increase for vascular grafts as treatment options for severe cardiovascular disease, significant research has begun to explore options beyond traditional autologous grafts. Artificially engineered vascular grafts have proven difficult to adequately match necessary material properties while maintaining long-term patency [41], so recent efforts have explored the use of decellularized blood vessels as a scaffold for vascular graft procedures [42–46]. Since decellularization nominally ameliorates immunogenicity by removing all cellular material, leaving only the ECM components responsible for passive mechanical properties, graft options need not be limited to human vessels. The most pressing need for improved graft options lies in small-diameter artery grafts, so decellularized conduit arteries of smaller mammals represent a potential avenue of research for graft options in humans. In theory, given basic information about a graft destination–e.g., pressure, deformed inner radius, and compliance–a clinician could reference a catalog of mechanically characterized non-human vessels, such as the investigation herein, to identify the most mechanically compatible scaffold for that particular situation.

While we support the scientific merit of this investigation, we must also acknowledge certain limitations. First, we chose not to quantify residual strains via opening angles of fresh cross-sectional slices. Residual strains are known to normalize the transmural stress distribution in a given vessel but can be difficult to measure reliably in the murine specimens. Also we assumed homogeneity of the vessel wall due to the relatively thin adventitial layer compared to a muscular artery or vein (see Fig 9). Further, due to the range of tissues used herein, animal ages could not be standardized; however, within a given species, all tested samples came from adult specimens of approximately equal age and size. For logistical reasons and to avoid confounding factors, we did not compare the specimens in their in vivo configurations but instead chose to evaluate all structural and mechanical metrics at common values of pressure and axial stretch. Furthermore, due to large variations in measured variables across species (e.g., axial force, vessel diameter), several hardware configurations were required to maintain fidelity of the collected data. Nevertheless, testing was consistent within a given species, all hardware was calibrated prior to testing, and protocols were consistent regardless of species or hardware.

## Supporting information

S1 Data Mechanical test data for mammalian carotid arteries (CCAs) subjected to inflation-extension testing. Circumferential results at λ_z_ = 1.5 and axial results at P = 100 mmHg for bovine, porcine, ovine, leporine, murine-rat, and murine-mouse subjects (n = 6 each). Values are mean ± SEM and the black filled cells indicate buckling.
(XLSX)
